# A novel prognostic biomarker DUSP6 promote the malignant progression of bladder cancer through mTOR mediated mitophagy

**DOI:** 10.3389/fonc.2025.1603069

**Published:** 2025-08-27

**Authors:** Jianbiao Huang, Chongwei Zhou, Zhaojun Yu, Zhen Song, Huanhuan Deng, Haichao Chao, Tao Zeng

**Affiliations:** ^1^ The Second Affiliated Hospital, Jiangxi Medical College, Nanchang University, Nanchang, Jiangxi, China; ^2^ The First Affiliated Hospital, Jiangxi Medical College, Nanchang University, Nanchang, Jiangxi, China

**Keywords:** bladder cancer, DUSP6, mitophagy, prognostic, mTOR

## Abstract

Bladder cancer (BC) is one of the most prevalent urinary malignant tumors that is intricately regulated by molecular pathways. Multiple studies have demonstrated a clear association between DUSP6 and malignant tumor progression; however, its role and underlying mechanisms in BC remain unclear. Here, we found that DUSP6 exhibits significantly elevated expression in BC tissues compared with normal tissues and is strongly associated with poor overall survival. Transcriptomic analysis revealed a robust correlation between DUSP6 expression and mitophagy, a selective form of autophagy crucial for maintaining mitochondrial integrity. *In vitro* and *in vivo* experiments demonstrated that knockdown of DUSP6 reduces tumor invasion, migration, and proliferation ability while enhancing mitophagy in BC cells. Notably, the anti-malignant effects of DUSP6 knockdown were partially reversed by the mitophagy inhibitor cyclosporin A. Mechanistically, DUSP6 modulates mitophagy by increasing the phosphorylation status of mTOR, a central autophagy regulator, and DUSP6 knockdown-induced mitophagy was partially restored after treatment with mTOR activator MHY1485. Our findings indicate that high DUSP6 expression promotes BC progression by inhibiting mTOR-mediated mitophagy, leading to a poor prognosis for BC patients. These insights suggest DUSP6 as a potential therapeutic target in the treatment of BC.

## 1 Introduction

Bladder cancer (BC) is one of the most common malignant tumors worldwide (1) with approximately 400,000 new cases and about 170,000 deaths every year (2). BC can be categorized into muscle-invasive bladder cancer (MIBC) and non-muscle-invasive bladder cancer (NMIBC) (3). Although there have been notable advancements in treat patients with NMIBC, more than 60% of individuals will still recurrence and more than 20% will develop into MIBC (4, 5). Thus, there is an imperative need for discovering an ideal novel target of therapeutic intervention in BC.

Dual-specificity phosphatases (DUSPs) serve as an inhibitor of the mitogen-activated protein kinase (MAPK) pathway (6), regulated cellular development and proliferation (6, 7). DUSP6, a protein of the DUSP family, regulating cellular functions through the dephosphorylation of extracellular signal-regulated kinase (ERK) (8). In recent years, roles of DUSP6 in tumor development are being gradually discovered (9–13). Ovarian cancer tissues have shown elevated levels of DUSP6, and suppress it expression can enhance the responsiveness to chemotherapy drugs in Ovarian cancer (13). At leukemia, high DUSP6 can activating Janus kinase 2 (JAK2) signaling pathway to promote tumor progression (9). But DUSP6 plays a tumor suppressor in endometrial cancer, low DUSP6 levels activating the ERK pathway to promote tumor progression (11).

Mitophagy is a specific type of autophagy that preserves the quality and quantity of mitochondria by selectively eliminating the depolarized, deceased, or redundancy mitochondria (14, 15). Mitochondrial malfunction, which is strongly linked to the development of numerous disorders, such as cancer, Alzheimer’s disease, sickle cell disease (14). *Monica* et al. found the buildup of malfunctioning mitochondria can lead to the formation of tumors (15). Research have shown that NIX-mediated mitophagy can promote the advancement of pancreatic cancer (16). Mitophagy, a process by which damaged mitochondria are degraded and recycled, is intricately associated with the regulation of cancer cell function and tumorigenesis (17). Thus, targeting mitophagy to cure cancer seems promising.

Elevated expression of DUSP6 was found in BC and correlated with overall survival. The hypothesis that DUSP6 mediates mitophagy, derived from single-cell seq data analysis, was experimentally validated both *in vitro* and *in vivo*. Additionally, we found DUSP6 influences Mitophagy through the mTOR pathway. Our results may provide a novel prognostic biomarker and potential therapeutic target for BC.

## 2 Materials and methods

### 2.1 Data sources

**Figure 1:**
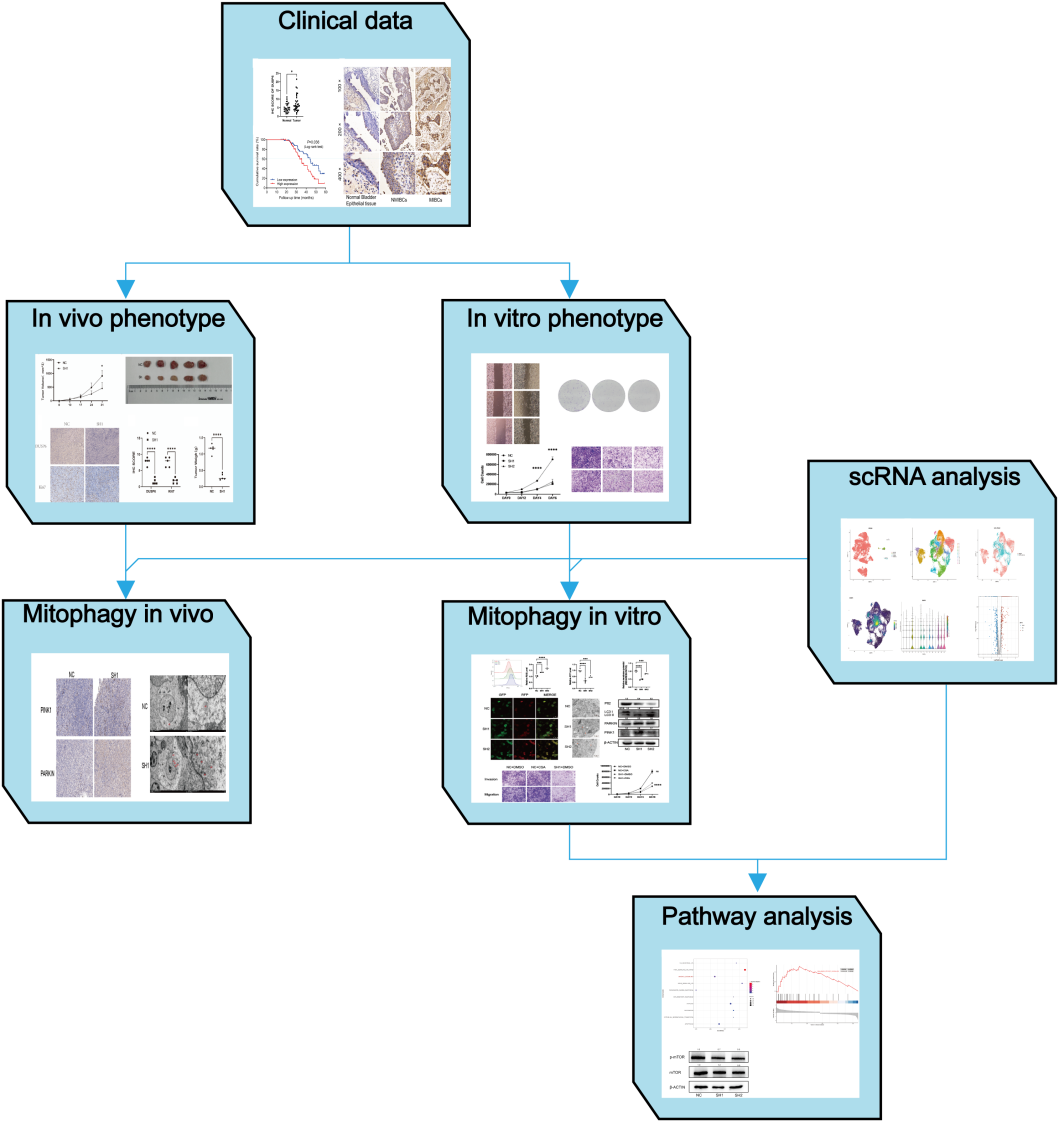
The brief flowchart of this research.

The Single-cell sequencing (scRNA-seq) data of BC (GSE135337) was download from the Gene Expression Omnibus database (GEO) (https://www.ncbi.nlm.nih.gov/geo/). TCGA-BLCA dataset download from The Cancer Genome Atlas Program (TCGA) (portal.gdc.cancer.gov). The flow chart of this study is shown in **Figure 1**.

### 2.2 Single cell analysis

ScRNA-seq were de-batched using the R package “harmony” (1.2.0) function “harmony”, space was further projected into a 2-dimensional space for visualization by Uniform Manifold Approximation and Projection (UMAP, R package: Seurat 4.4.4), then using resolution=0.5 for cell clustering. and labelled with broadly marker epithelial (EPCAM, KRT13, KRT7, KRT18), fibroblast (DCN, COL3A1, COL1A1), immune (PTPRC), or endothelial (PLVAP, VWF, CLDN5) using a panel of marker genes gleaned from the literature.

DEGs were identified using the function “FindMarkers” with a threshold of |log2(FC)| > 0.25 and an adjusted P value < 0.05.

### 2.3 Enrichment analysis

Kyoto Encyclopedia of Genes and Genome (KEGG), Gene Ontology (GO) enrichment analysis and Gene Set Enrichment Analysis (GSEA) were performed for differential genes through the R package “clusterProfiler” (4.10.0), and the enrichment results with P value < 0.05 were selected.

### 2.4 Clinical samples and immunohistochemistry

A total of 122 BC tissues and 34 non-tumor bladder tissues were collected from BC patients at The Second Affiliated Hospital of Nanchang University between 2019 and 2023. All patients did not receive radiotherapy, chemotherapy, or immunotherapy prior to surgical treatment and were pathologically diagnosed with BC. All methods were carried out in accordance with relevant guidelines and regulations, and all experimental protocols were approved by the Ethics Committee of the Second Affiliated Hospital of Nanchang University (Approval No.: IIT- 2024-228). Confirming that informed consent was obtained from all subjects or their legal guardian.

Collected tissues were fixed with 4% paraformaldehyde for 4 hours, sliced into 3.5 μm after paraffin embedding. Then after deparaffinization and rehydration, heat-induced antigen retrieval with sodium citrate was performed for 20 min. Followed by quenching of endogenous peroxidase activity though 3% hydrogen peroxide for 5 min. Primary antibody incubation with DUSP6 (Santa Cruz Biotechnology, sc-377070, 1:50), KI-67(Abmart Inc, TW0001, 1:50), PINK1(Santa Cruz Biotechnology, sc-517353, 1:50), PARKIN (WANLEIBIO, WL02512, 1:100) was performed overnight at 4°C. Flowed by hematoxylin, biotinylated secondary antibody, and 3,3’-diaminobenzidine (DAB) solution (Zhongshan Golden Bridge Biotechnology Co Ltd, ShangDong, China) were then used for standing.

### 2.5 Cell cultivation and stable cell line establish

The human BC cell line T24 was obtained from the China Academia Sinica Cell Repository (Shanghai, China), and the UC3 cell line was acquired from Procell (Wuhan, China) with STR profiling. T24 cells were cultured in DMEM (Servicebio, Wuhan, China), and UC3 cells were cultured in MEM (Servicebio, Wuhan, China), both supplemented with 10% fetal bovine serum (Excell, Suzhou, China). Plasmids LV-shNC, LV-shDUSP6-1, LV-shDUSP6-2, and LV-shDUSP6-3 (SH1: GGAGAACGCAGGAGAGTTTAAA; SH2: GCTGTGGTGTCTTGGTACATTG; SH3: AAACTGTGGTGTCTTGGTACAT) purchase from Qingke (Beijing Qingke Biotechnology Co., Ltd.) were used to construct stable cell line.

A total of 20 μg plasmids PxpAx2, PMD2g, and PLVX were mixed at ratio of 3:1:4 in 500 μl of serum-antibiotic-free DMEM. 50 μg of PEI (Yeasen Biotechnology (Shanghai) Co., Ltd.) was diluted in 500 μl of the same medium. The two solutions were mixed, incubated at room temperature for 20 minutes, and added to 293T cells in a 10 cm dish. After 18 hours, the medium was replaced with complete medium, and the cell supernatant was collected at 48 and 72 hours for subsequent cell infection.

Then bladder cancer cells in logarithmic growth phase were transfected by lentivirus, following by culturing in 1640/MEM/DMEM medium with 10% FBS in a 6-well dish. Puromycin (2 μg/μl) was added for selection when the cell density reached 90%, and the stable colonies will be amplified after 10–14 days. The overexpression/knockdown efficiency of TEAD4 was evaluated by qPCR and western blot.

### 2.6 Western blot

The cell was lysed in RIPA buffer (Epizyme Biomedical Technology, shanghai, China), and electrophoresis was using 10% SDS-PAGE (Epizyme Biomedical Technology, shanghai, China) then transferred protein into PVDF membranes (Merck Millipore Burlington, USA), after 1.5 h block incubate with first antibodies: DUSP6 (Santa Cruz Biotechnology, sc-377070, 1:200), PINK1 (Santa Cruz Biotechnology, sc-517353 1:500),P62 (Abmart Inc, T55546, 1:5000),LC3 (Abmart Inc, T55992, 1:500), mTOR (Abmart Inc, T56571 1:1000), P-mTOR (Abmart Inc, T56571 1:1000), PARKIN (WANLEIBIO, WL02512, 1:1000), ACTIN (Servicebio, GB113225-100, 1:1000) for a night at 4°C. Next day, incubated with second antibody (Servicebio, GB23301 1:10000, GB23303 1:10000) at room temperature for 1.5 h. Finally, the blots were incubated with ECL reagents (Servicebio, Wuhan, China) and visualized using Image J software.

### 2.7 Real-time quantitative PCR

Real-time quantitative PCR assay using Universal SYBR Green qPCR Master Mix (Servicebio, Wuhan, China). The primer purchased from Sangon (Sangon Biotech (Shanghai) Co., Ltd.) and sequences are as follows:DUSP6: Forword:5′- GAACTGTGGTGTCTTGGTACATT -3′; Reverse: 5′- GTTCATCGACAGATTGAGCTTCT -3′; β-ACTIN: Forword: 5′ - CATGTACGTTGCTATCCAGGC -3 ′; Reverse: 5′- CTCCTTAATGTCACGCACGAT -3 ′. The program as follows: 1. Initial denaturation for 3 min. 2. Denaturation for 20 second. 3. Annealing and extension for 20 seconds.

### 2.8 Colony formation assay

500 cells were seeded in 6-well plates with culture medium containing 10% FBS and cultured for 1–2 weeks. The cell colonies were fixed with 4% paraformaldehyde for 30 minutes, then staining with 0.1% crystal violet for an additional 30 minutes. Finally, images were captured using a high-definition digital camera and analyzed using ImageJ software.

### 2.9 Cell proliferation assay

Cells were seeded into 12-well plates at a density of 10,000 cells per well and maintained at 37°C in an atmosphere of 5% CO2. At 2, 4, and 6 days, the cells were digested, resuspended in total 500 µL of medium, and the cell density was measured using a flow cytometer.

### 2.10 Migration and invasion assays

*Transwell* chambers (Labselect, 8 μm pore size, Anhui, China) with or without Matrigel was used to evaluate the invasion or migration ability of BLCA cells. The procedure involved placing 2 × 10^5 cells into the upper compartment, which was filled with 200 μL of a serum-free medium. In the lower compartment, 500 μL of medium supplemented with 10% FBS was introduced. Following an incubation period of 16 to 24 hours at 37°C, the Transwell chambers were rinsed with PBS and treated with 4% paraformaldehyde for about 15 minutes. Subsequently, the cells on the upper surface of the membrane were wiped off using a cotton swab, and the remaining cells were stained with crystal violet for approximately 15 minutes at ambient temperature. Finally, the membranes were rinsed again in PBS, allowed to air-dry, and then documented with photographs.

### 2.11 Wound healing assay

A 6-well plate was used for cell seeding when conducting the wound-healing assay. A 200μL sterile plastic pipette was employed to create a scratch in the cell monolayer. Following this, the cells were placed in a medium devoid of FBS for cultivation. Photographs were taken at two time points, 0 and 24 hours, utilizing an electron microscope. The cells’ migratory capacity was assessed through the quantification of the wound area’s dimensional alterations.

### 2.12 *In vivo* studies

Four-week-old male nude mice were purchased from SPF (SPF biotechnology, Beijing) biotechnology. Transfected cells were subcutaneously injected (1 × 10^6 in 200 μL Matrigel) in 5 mice per group. Four weeks later, after anesthesia with sodium phenobarbital, the mice were euthanized, and the tumor tissues were harvested for subsequent experiments. And all methods were carried out in accordance with relevant guidelines and regulations, and all experimental protocols were approved by Institutional Animal Care and Use Committee, Nanchang University (Approval No.: NCULAE-20221031184).

### 2.13 Electron microscopy

Cells or tissue were fixed in 2.5% glutaraldehyde (Servicebio, Wuhan, China) at 4 °C for 4 h, pre-embedded in 1% agar to maintain integrity, washed with PBS, and thereafter post-fixed for 2 h in the presence of a 1% OsO4 buffer at 4°C. After washing, cells were dehydrated in graded ethanol concentrations and embedded using Epon812 epoxy resin. Ultrathin sections (90 nm) were obtained on copper grids, double-stained with 0.2% lead citrate and 1% uranyl acetate, followed by examination by HT7800 transmission electron microscopy (Hitachi, Japan).

### 2.14 Mitochondrial function and autophagy assays

ROS: Diluted DCFH-DA (Beyotime, Shanghai) with serum-free culture medium (1:1000). After trypsinizing and counting the cells, take out 500,000 cells, centrifuge and remove the medium, resuspend the cells with diluted DCFH-DA. Incubate at 37°C cell culture incubator for 20 minutes. Then using flow cytometer with an excitation wavelength (Ex) of 488nm and an emission wavelength (Em) of 525nm to detect the fluorescence.

ATP: Dilute the ATP detection reagent (Beyotime, Shanghai) with diluent at ratio of 1:4. and add 200 μL of lysis solution to 2 million cells. After lysing, centrifuge at 12,000g for 5 minutes at 4°C, the supernatant was used for subsequent assay. Add 100 microliters of ATP working solution to 96-well plate, stand at room temperature for 3–5 minutes before adding 20 microliters of lysate supernatant. At last, measure the relative light units (RLU) though multifunctional microplate reader.

Mitochondrial membrane potential: Dilute JC-1(Beyotime, Shanghai) with staining buffer at ratio of 1:200, add 500 microliters culture medium and 500 microliters diluted working solution to resuspend the cells. then Incubate it at 37°C for 20 minutes and centrifuge at 600g for 3 minutes at 4°C wash the cells three times with PBS before resuspending in buffer. Finally, measure the fluorescence intensity of JC-1 monomers (Ex:485 Em:535) and JC-1 aggregates (Ex:550 Em:600) though flow cytometer.

Autophagy: Trypsinizing and seeding cells to ensure that the cell density is approximately 50% in the next day. Discard the old culture medium and add fresh medium The mCherry-GFP-LC3B (Genechem, Shanghai) adenovirus added according to MOI = 10. After a two-day incubation, the cells were imaged by confocal microscope to visualize autophagy flux.

### 2.15 Reverses assay

Before being collected for subsequent experiments, cells were pretreated with complete medium containing 0.5 μM mitophagy inhibiter ciclosporin A (CSA, MedChemExpress) for 24 hours.

Cells were treated with a complete medium containing 1.5 μM mTOR agonist MHY1485 (MedChemExpress) for 12 hours, after that they were collected for subsequent experiments.

### 2.16 Statistical analysis

Unless otherwise stated, assays were repeated thrice, and all data showed as independent data points. SPSS 25.0 was used for data analyses. For quantitative data, normality testing is used to ascertain whether the data is normally distributed, and then a two-tailed Student’s t-test or non-parametric test is utilized. The χ2 test or Fisher’s exact test is conducted for qualitative data. P < 0.05 is accepted as being statistically significant.

## 3 Results

### 3.1 Increased DUSP6 expression were related to more aggressive BC and a worse prognosis

**Figure 2:**
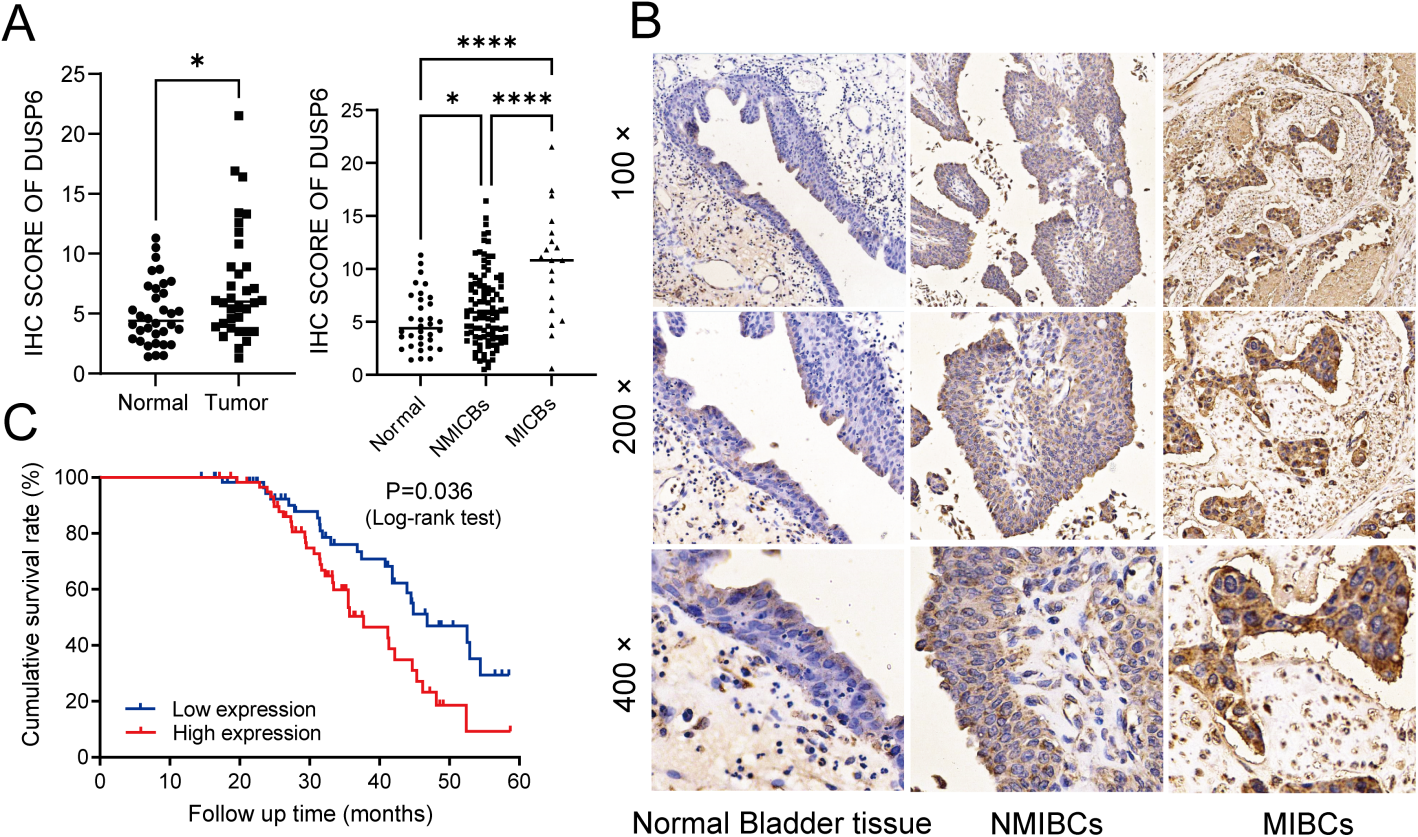
Investigating the associations among DUSP6 expression, clinicopathological traits, and prognosis of the BC patients. **(A)** left: Expression of DUSP6 in bladder cancer and non-tumor tissue. Right: Expression of DUSP6 in non-tumor tissue, NMIBCs and MIBCs. **(B)** IHC staining of DUSP6 in non-tumor bladder tissues, non-muscle-invasive bladder cancer (NMIBC) tissues and muscle-invasive bladder cancer (MIBC) tissues. **(C)** Kaplan-Meier survival analysis of DUSP6 for overall survival. *P<0.05, ****P<0.0001.

In our previous study, while RAB14 was knocked down, we found the expression of DUSP6 was significantly elevated in BC cells (18). In order to explore the expression of DUSP6 in BC tissues, 122 tumor tissues and 34 non-tumor tissues were used for immunohistochemical (IHC) analysis. The findings indicated that the immune scores of tumor tissues were higher than those of non-tumor tissues (**Figure 2A**). Additionally, the staining intensity of MIBC was observed to be stronger than NMIBC (**Figures 2A, B**). Then, the results of the clinical correlation analysis indicated that the expression level of DUSP6 was positively correlated with T stage, N stage, and grade (**Table 1**). However, no correlation was observed between DUSP6 expression and age, sex, M stage, or tumor differentiation (**Table 1**).

**Table 1:** Relationship between DUSP6 expression and clinicopathological features of patients with bladder cancer.

Clinicopathological	n	DUSP6		χ2	*P*
		Low	High		
Gender					
male	84	26	58	3.058	0.803
female	38	18	20		
Age (years)					
<60	47	13	34	2.343	0.126
>60	75	31	44		
T					
T1-T2	41	20	21	4.330	0.037
T3-T4	81	24	57		
N					
N0	29	16	13	6.023	0.014
N1-N2	93	28	65		
M					
M0	45	12	33	2.732	0.098
M1	77	32	45		
TNM					
I~II	36	20	16	8.413	0.004
III~IV	86	24	62		
Differentiation degree					
High-medium	39	18	21	2.530	0.112
poorly	83	26	57		

A five-year follow-up survey of 122 BC patients revealed that the median survival time for patients with low DUSP6 expression was 46.8 weeks, which was significantly longer than the 37.7 weeks observed for patients with high DUSP6 expression. Kaplan-Meier survival analysis highlighting the potential of DUSP6 as a prognostic marker for patient survival in BC (*p* < 0.05). (**Figure 2C**).

### 3.2 Silencing DUSP6 inhibits BC cells proliferation, invasion and migration ability

**Figure 3:**
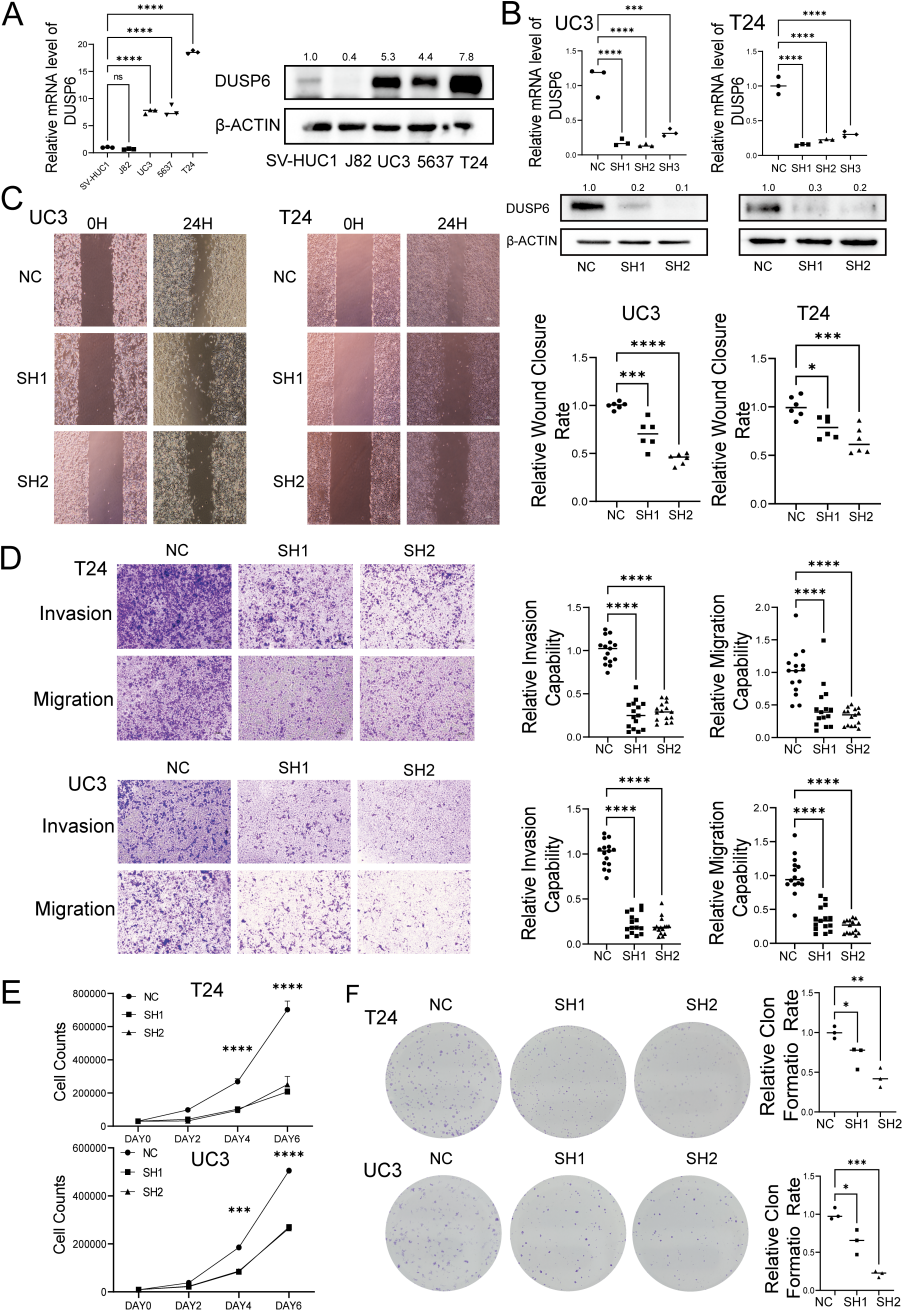
Effect of knock-down DUSP6 on BC cell line *in vitro* experiments. **(A)** Basal DUSP6 expression of mRNA and protein in bladder cancer (BC) cell line and normal bladder epithelial cell line. **(B)** Western blot and RT-qPC analysis of DUSP6 expression level in the BC cells transfected with LV-shDUSP6 and LV-NC. **(C)** Wound-healing assays were performed to assess the migration capacity of the DUSP6 knockdown cells. **(D)** Transwell assays were performed to assess the migratory and invasion capacity of the DUSP6 knockdown cells. **(E)** Cell proliferation assays were performed to assess the proliferation capacity of the DUSP6 knockdown cells. **(F)** Colony assays were performed to assess the clone formation capacity of the DUSP6 knockdown cells. *P<0.05, **P<0.01, ***P<0.001, ****P<0.0001, ns, not significant.

The basic expression level of DUSP6 in BC cell lines was examined by qRT-PCR and Western blot. The results demonstrated that the mRNA and protein expression levels of DUSP6 in UC3, 5637, and T24 were significantly higher than those in the normal cell line SV-HUC-1(**Figure 3A**). In accordance with the aforementioned results, UC3 and T24 cells exhibiting elevated endogenous DUSP6 expression were selected for the *in vitro* knockdown assay. The knockdown efficiency was determined by qRT-PCR, which revealed that shRNA#1 and shRNA#2 exhibited superior knockdown efficiency compared to shRNA#3 (p<0.001, **Figure 3B**). Then, we investigated the proliferative, invasive, and metastatic capabilities of stable cell lines with DUSP6 knockdown (shRNA#1&shRNA#2). The results of Wound healing and Transwell assays found DUSP6 knockdown markedly hindered the invasive and migratory activities of BC cells (**Figures 3C, D**). Additionally, silencing DUSP6 also suppressed proliferation (**Figure 3E**) and colony formation (**Figure 3F**).

### 3.3 Knockdown of DUSP6 inhibit tumor growth *in vivo*

**Figure 4:**
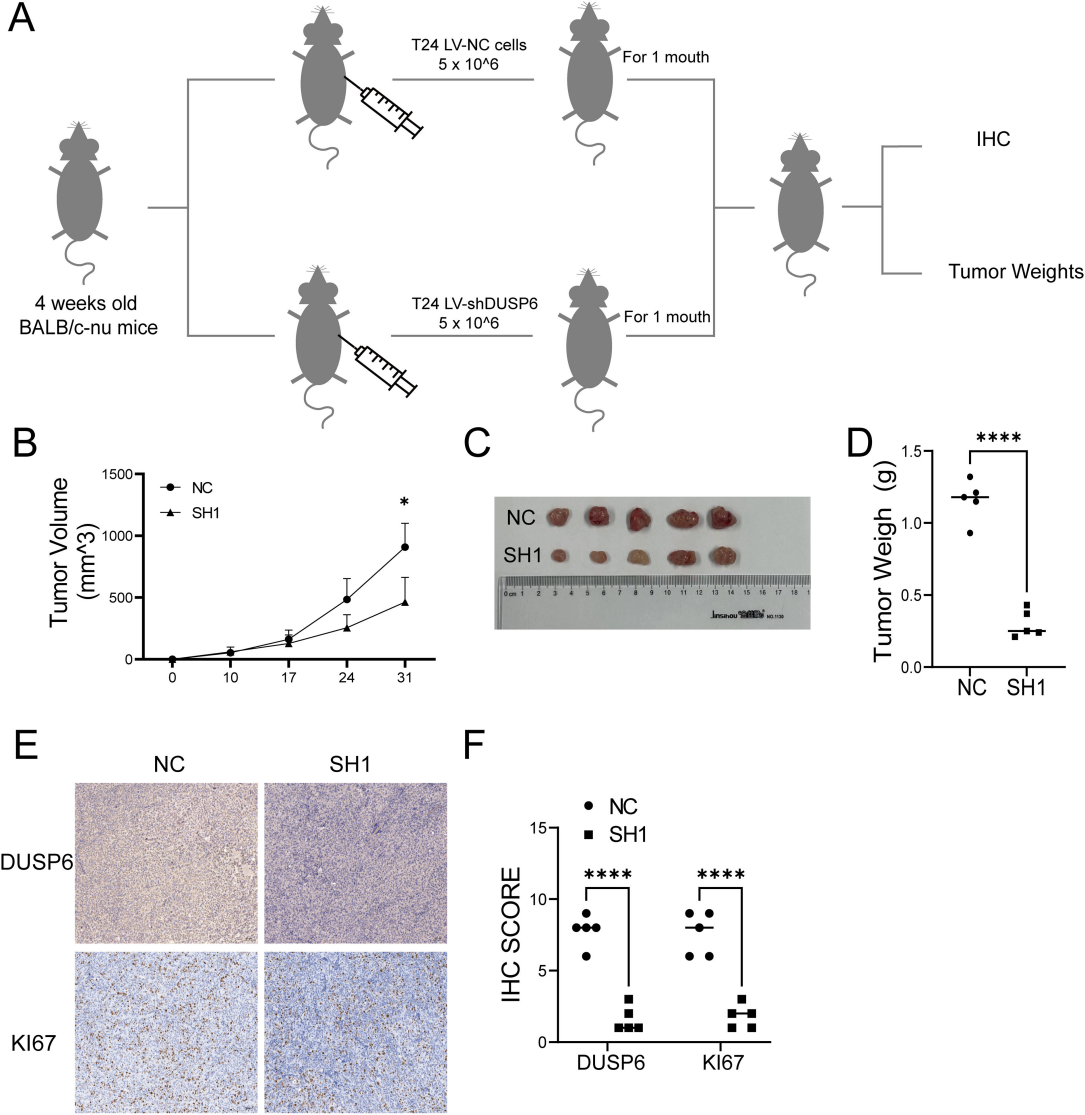
Effect of knock-down DUSP6 on BC cell line *in vivo* experiments. **(A)** Schematic diagram of xenograft model of nude mice. **(B)** Volume change of in xenograft tumor in nude mice within 1 month. **(C)** The general appearance of the xenograft tumor tissue from the LV-shDUSP6 and LV-NC groups. **(D)** Discrepancy tumor weight between LV-shDUSP6 and LV-NC groups. **(E, F)** IHC and staining of DUSP6 and KI67 expression levels in LV-shDUSP6 and LV-NC groups. *P<0.05, ****P<0.0001.

In the *in vivo* experiments utilizing a xenograft tumor model of nude mouse with subcutaneously injected T24 cells (**Figure 4A**), both the tumor volume and weight were decreased in the DUSP6 knockdown group compared to the control group (**Figures 4B–D**). Immunohistochemical examination of Ki67 staining demonstrated that DUSP6 knockdown inhibited tumor cell growth *in vivo* (**Figures 4E, F**).

### 3.4 DUSP6 is associated with mitochondrial function and mitophagy

**Figure 5:**
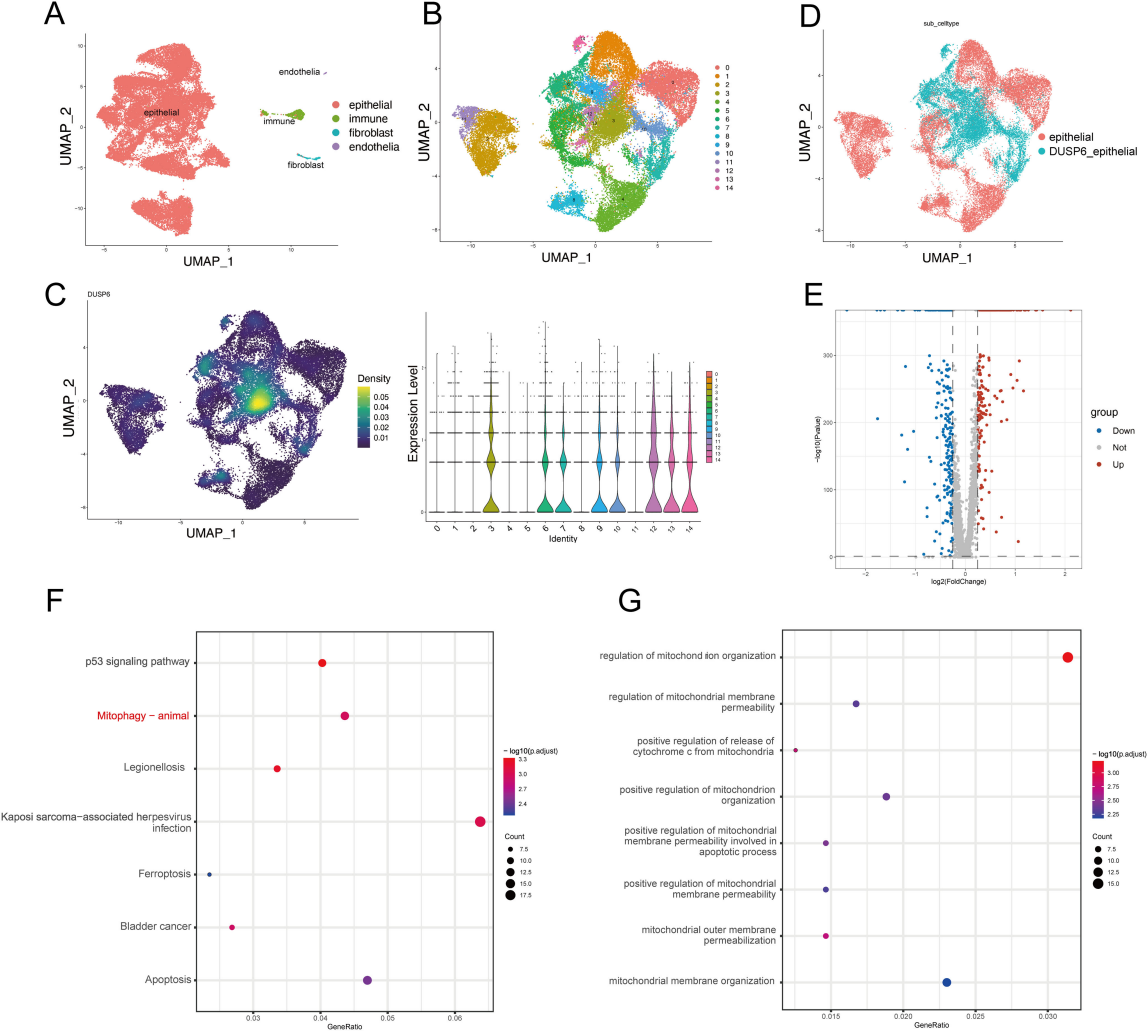
DUSP6 expression may be associated with mitophagy. **(A)** The UMAP plot of the single-cell seq data annotation showed epithelial cells in red, immune cells in green, fibroblasts in blue, and endothelial cells in purple. **(B)** The UMAP plot showed clustering of epithelial cells. Epithelial cells are divided into 15 clusters. **(C)** DUSP6 expression in epithelial cells of tumor tissues is illustrated, with expression density plot at left and violin plot at right of DUSP6. **(D)** The UMAP plot displayed that epithelial cells were separated into DUSP6+ epithelium and DUSP6- epithelial based on their expression. **(E)** The volcano plot illustrated the differential expression genes between DUSP6+ epithelial cells and DUSP6-epithelial cells. **(F)** The KEGG enrichment analysis for differential genes was performed. **(G)** The GO enrichment analysis for differential genes was performed.

In order to investigate the mechanism by which DUSP6 influences the malignant progression of BC. We preprocessed and annotated single-cell seq data and found DUSP6 show a high expression in endothelial cells and certain epithelial clusters (**Figure 5A**, **Supplementary Figures 1A-D**). Then the epithelial cells were extracted (**Figure 5B**). Cluster 3, 6, 7, 9, 10, 12, 13, 14 displayed higher expression levels of DUSP6 and were defined as DUSP6+ epithelial cells (**Figures 5C, D**). Subsequently, a differential expression analysis was conducted on DUSP6+ epithelial cells in comparison to other epithelial cells, resulting in the identification of 534 differentially expressed genes (DEGs) (**Figure 5E**, **Supplementary Table 1**). An KEGG pathway enrichment analysis of these DEGs revealed that the enrichment pathways were p53 signaling pathway, mitophagy pathway, ferroptosis and apoptosis (**Figure 5F**). The biological process analysis further showed DUSP6 may influence the mitochondrial function (**Figure 5G**). In the independent external validation set TCGA, we also found that DUSP6 is associated with mitophagy and multiple mitochondrial functions (**Supplementary Figures 2A-C**, **Supplementary Table 2**).

These findings demonstrate that DUSP6 is associated with multiple tumor-related processes, including the p53 signaling pathway, mitophagy, ferroptosis, and apoptosis. Among these, mitophagy was selected for further analysis due to its particular relevance to DUSP6.

### 3.5 DUSP6 promotes BC progression by inhibiting mitophagy

**Figure 6:**
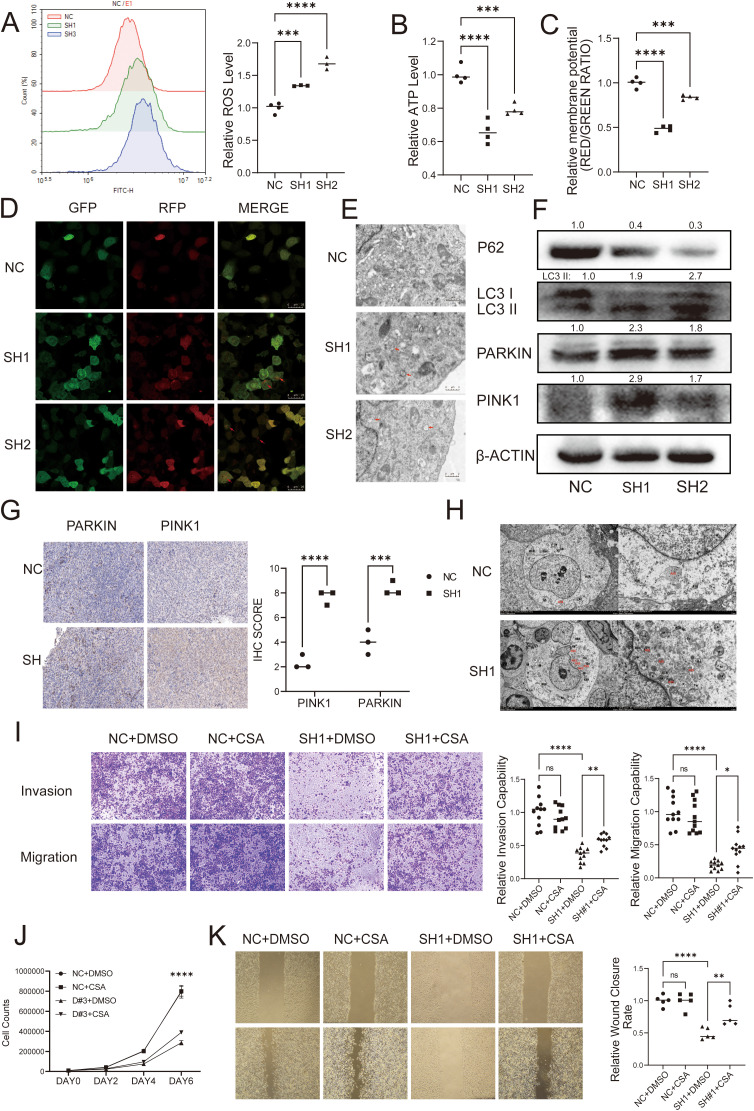
DUSP6 promotes BC progression through inhibiting mitophagy. **(A)** The ROS level was detected by Flow cytometric analysis. **(B)** Microplate reader detected the cellular ATP levels. **(C)** The mitochondrial membrane potential levels was detected by JC-1 assay. **(D)** Confocal laser microscopy showed the autophagy flux in T24 cells with DUSP6 knockdown. Red arrows point to autophagy lysosomes. **(E)** Transmission electron microscopy showed mitophagy in T24 cells with DUSP6 knockdown. Red arrows point to autophagy lysosomes. **(F)** Western Blot analyzed the relative expression levels of P62, LC3, PARKIN, and PINK1 in T24 cells transfected with LV-NC or LV-shDUSP6. **(G)** IHC was used to detect the expression of PARKIN and PINK1 in mice tumor tissue. **(H)** Transmission electron microscopy observed mitophagy in mouse tumor tissues. (N, nucleus; M, mitochondria; ASS, autophagic lysosome; AP, autophagosome; Go, Golgi apparatus; RER, rough endoplasmic reticulum. **(I)** After treated with CsA for 24h, transwell assay assessed the invasion and migration ability of DUSP6 knockdown cells. **(J)** After treated with CsA for 24h, Cell proliferation assay assessed the invasion and migration ability of DUSP6 knockdown cells. **(K)** After treated with CsA for 24h, Wound-healing assays assessed the migration ability of DUSP6 knockdown cells. *P<0.05, **P<0.01, ***P<0.001, ****P<0.0001, ns, not significant.

In order to verify the relationship between DUSP6 and autophagy, we first analyzed the oxygen species (ROS), adenosine triphosphate (ATP), and mitochondrial membrane potential levels in DUSP6 knockdown cells. The results showed that shRNA-DUSP6 knockdown in BC cells result in significantly increase of intracellular ROS and decrease in ATP levels (**Figures 6A, B**). We also found a significant decrease in mitochondrial membrane potential in DUSP6 knockdown stable cells (**Figure 6C**). These results indicated that mitochondrial dysfunction occurred after DUSP6 knockdown. Additionally, enhanced autophagic flux and increased the number of autophagosomes within the cells were also found in DUSP6 knockdown cells (**Figures 6D, E**). This was accompanied by changes in the expression of mitophagy markers. The result of western blot indicated silencing DUSP6 could down-regulate P62 and up-regulate PARKIN, PINK1 and LC3II/I (**Figure 6F**). Then, we performed IHC staining to analyze PINK1 and PARKIN expression in xenograft tumor sample from nude mice. Similar to our previous results, it also demonstrated a significant elevation in PINK1 and PARKIN expression in tumor tissues subjected to DUSP6 knockdown (**Figure 6G**). The results of transmission electron microscopy provided further confirmation of the presence of elevated mitophagy (**Figure 6H**).

To elucidate whether mitophagy plays a role in the regulation of the malignant phenotype of invasion, metastasis, and proliferation in BC by DUSP6, we pre-treated cells with the mitophagy inhibitor CsA with 0.5 μM for 24 hours and performed functional phenotype assays. the results showed the CsA reversed the effects of DUSP6 knockdown on cell migration, invasion (**Figures 6I, K**), and proliferation partially (**Figure 6J**).

The findings indicate that DUSP6 may contribute to the malignant progression of BC by influencing mitophagy.

### 3.6 DUSP6 may influence mitophagy through activating the mTOR pathway

**Figure 7:**
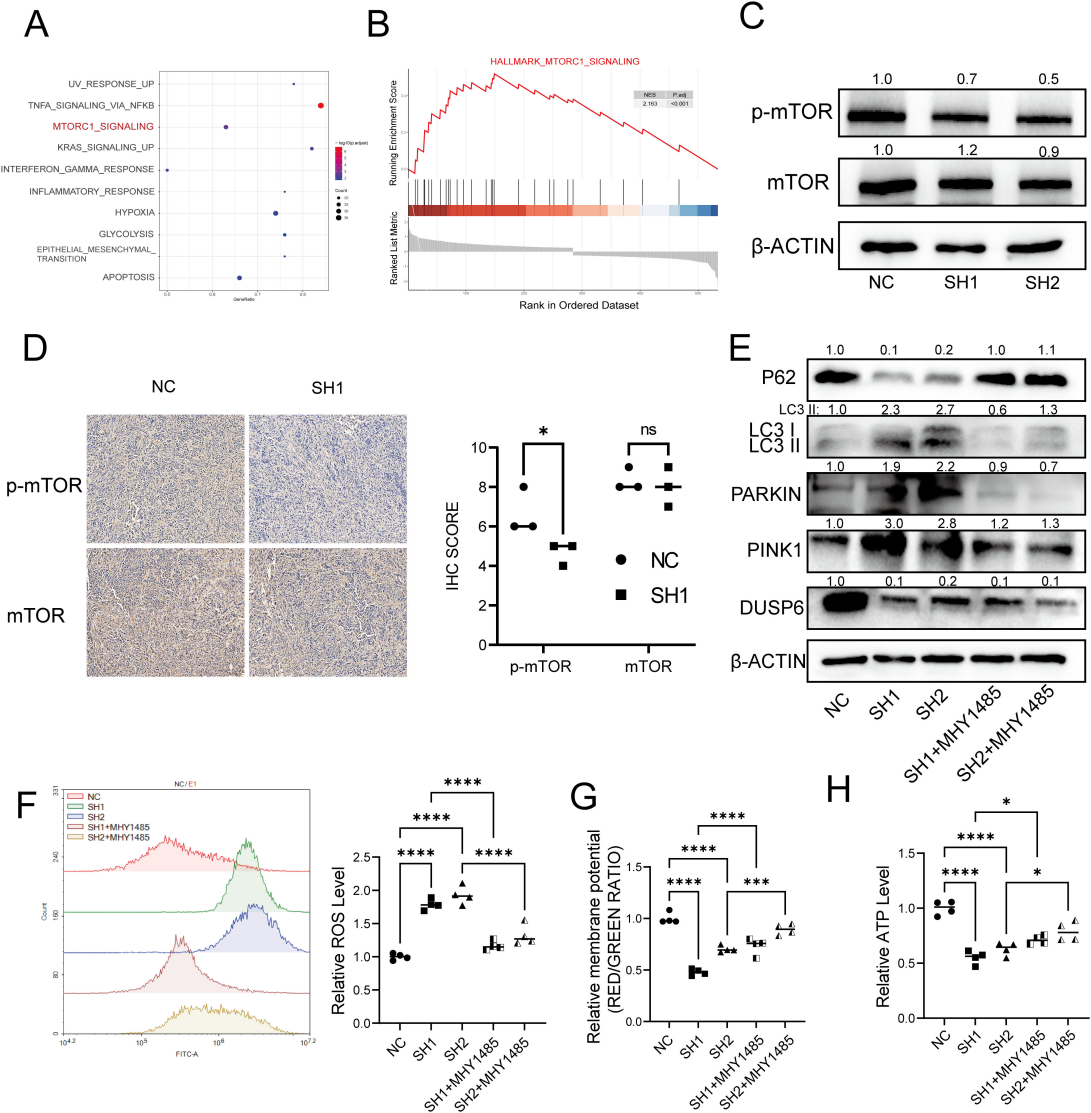
DUSP6 knockdown inactivated the mTOR signaling pathway. **(A)** Dot plot of GSEA enrichment analysis result. **(B)** GSEA enrichment analysis of differential expressed genes. **(C)** Western Blot analyzed the mTOR and p-mTOR protein levels in DUSP6 knockdown cells. **(D)** Expression levels of mTOR and p-mTOR in xenograft samples. **(E)** Changes in mitophagy markers after 12h treatment with MHY1485 in T24 LV-shDUSP6 cells. **(F)** The ROS level after treat with MHY1485 for 12h. **(G)** The ATP level after treat with MHY1485 for 12h. **(H)** The mitochondrial membrane potential levels after treat with MHY1485 for 12h. *P<0.05, ***P<0.001, ****P<0.0001, ns, not significant.

To investigate the potential mechanisms through which DUSP6 influences mitochondrial autophagy, we conducted Gene Set Enrichment Analysis (GSEA). The results indicated the existence of a notable positive correlation between DUSP6 and the mTOR pathway. (**Figures 7A, B**). Several studies have demonstrated that this pathway is associated with mitochondrial biogenesis and mitochondrial function (19–23). To substantiate the hypothesis that DUSP6 regulates the mTOR pathway, western blot analysis was conducted to examine alterations in mTOR phosphorylation levels in DUSP6 knockdown stable cells. The results demonstrated a reduction in the levels of mTOR phosphorylation (**Figure 7C**), Similarly, in the xenograft tumor samples, the p-mTOR level was found to be reduced in the sh-DUSP6 group (**Figure 7D**).

To further confirm whether DUSP6 mediates mitophagy in bladder cancer through the mTOR pathway, we pre-treated DUSP6 knockdown cells with medium containing 1.5 μM of mTOR agonist MHY1485 for 12 hours, and observed that the level of mitophagy was decreased (**Figure 7E**). Similarly, after treatment, ROS levels were decreased (**Figure 7F**) while mitochondrial membrane potential (**Figure 7G**) and ATP levels (**Figure 7H**) were restored in DUSP6 knockdown cell line. There results indicate that the DUSP6 mediates mitophagy through mTOR pathway in bladder cancer.

## 4 Discussion

Previous study has demonstrated that DUSP6 have either inhibitory or promotive effects on tumor progression in different types of malignancies (8, 10–13). However, its precise role in BC has not been documented before. Our study contributes to the comprehension of DUSP6 in BC by demonstrating that DUSP6 expression is higher in tumor tissues compared to non-tumor tissues. Furthermore, its expression rises with tumor malignancy increases, and patient prognosis is negatively correlated with DUSP6 expression level. These findings were subsequently confirmed through further investigations conducted *in vitro* and *in vivo*.

With the advent of high-throughput sequencing in recent decades, our understanding of BC biology has been significantly advanced (24–26). Through the analysis of single-cell seq data, we have discovered that DUSP6 may be functionally linked to many mitochondrial functions in BC and our KEGG enrichment analysis showed that DUSP6 may also involve in mitophagy. The findings of the bioinformatics analyses provided a rationale for hypothesizing that DUSP6 may regulate mitophagy. To substantiate this conjecture, we subsequently conducted *in vitro* and *in vivo* experiments. In the assays designed to investigate the effect of DUSP6 on mitochondrial function, we observed that the down-regulation of DUSP6 expression resulted in a reduction in intracellular levels of ATP and mitochondrial membrane potential, accompanied by an increase in ROS levels. Subsequent western blot and electron microscopy assays demonstrated that mitophagy levels increased following DUSP6 downregulation. The results of the mitochondrial function and mitophagy experiments corroborate the conclusion derived from the single-cell seq data analysis, namely that DUSP6 plays a negatively regulatory role in mitophagy. The study of Tsai et al (27). demonstrated that inhibition of DUSP6 activity enhances autophagy flux in retinal pigment epithelium cells. The findings of our study are in agreement with this result. However, Tsai et al.’s study did not investigate the specific type of cellular autophagy affected by DUSP6, especially the mitophagy.

Several investigations have shown a correlation between the mTOR signaling pathway and levels of mitophagy (2–4). The mTOR pathway was strongly related with DUSP6-expressed genes in the KEGG enrichment analysis, suggesting that DUSP6 may regulate mitophagy in BC though mTOR pathway. We also found that DUSP6 knockdown decreased mTOR phosphorylation, suggesting that DUSP6 may activate the mTOR pathway in BC. Given the evidence from multiple studies indicating the involvement of the mTOR pathway in the regulation of mitophagy (19, 22, 23) and the findings of the present study, it is reasonable to infer that DUSP6 may inhibit mitophagy in BC by activating the mTOR pathway.

Due to limitations in technology and time, although our transcriptomic analysis revealed that DUSP6 is associated with several key cancer-related pathways or functions, such as p53, Wnt signaling pathway, ferroptosis, and apoptosis, we did not further investigate these relationships. Instead, we focused our subsequent research on what we consider the most critical aspect: mitophagy. Moreover, further investigation is needed to fully understand the mechanism by which DUSP6-regulated mitophagy contributes to the malignant advancement of BC. In order to tackle these concerns, we will carry out additional study in our future investigations.

To summarize, our results suggest that DUSP6 is a strong indicator of poor prognosis in BC. It achieves this by suppressing the process of mitophagy in BC cells through the inactivation of the mTOR pathway. Consequently, this leads to increased cell migration, invasion, and proliferation. These findings not only provide a reliable biomarker for predicting prognosis, but also indicate a possible target for therapeutic intervention in metastatic BC.

## Data Availability

All data download from the online database, all codes used in this study can be acquired from the first or corresponding author.

## Supplementary material

Supplementary Figure 1
**(A)** The UMAP plot before remove batch effect. **(B)** The UMAP plot of ‘harmony’ used to remove batch effect. **(C)** Cluster of scRNA-seq. **(D)** Dot plot show the marker genes and DUSP6 expression in every cluster.

Supplementary Figure 2
**(A)** Differential expression genes between DUSP6 high and low group in TCGA-BLCA dataset **(B)** The KEGG enrichment analysis for differential genes was performed. **(C)** The GO enrichment analysis for differential genes was performed.
