# Supplementary figures and images for: A novel prognostic biomarker DUSP6 promote the malignant progression of bladder cancer through mTOR mediated mitophagy
Source: Front Oncol. 2025 Aug 27;15:1603069. doi: 10.3389/fonc.2025.1603069 (PMC12420332; doi:10.3389/fonc.2025.1603069)

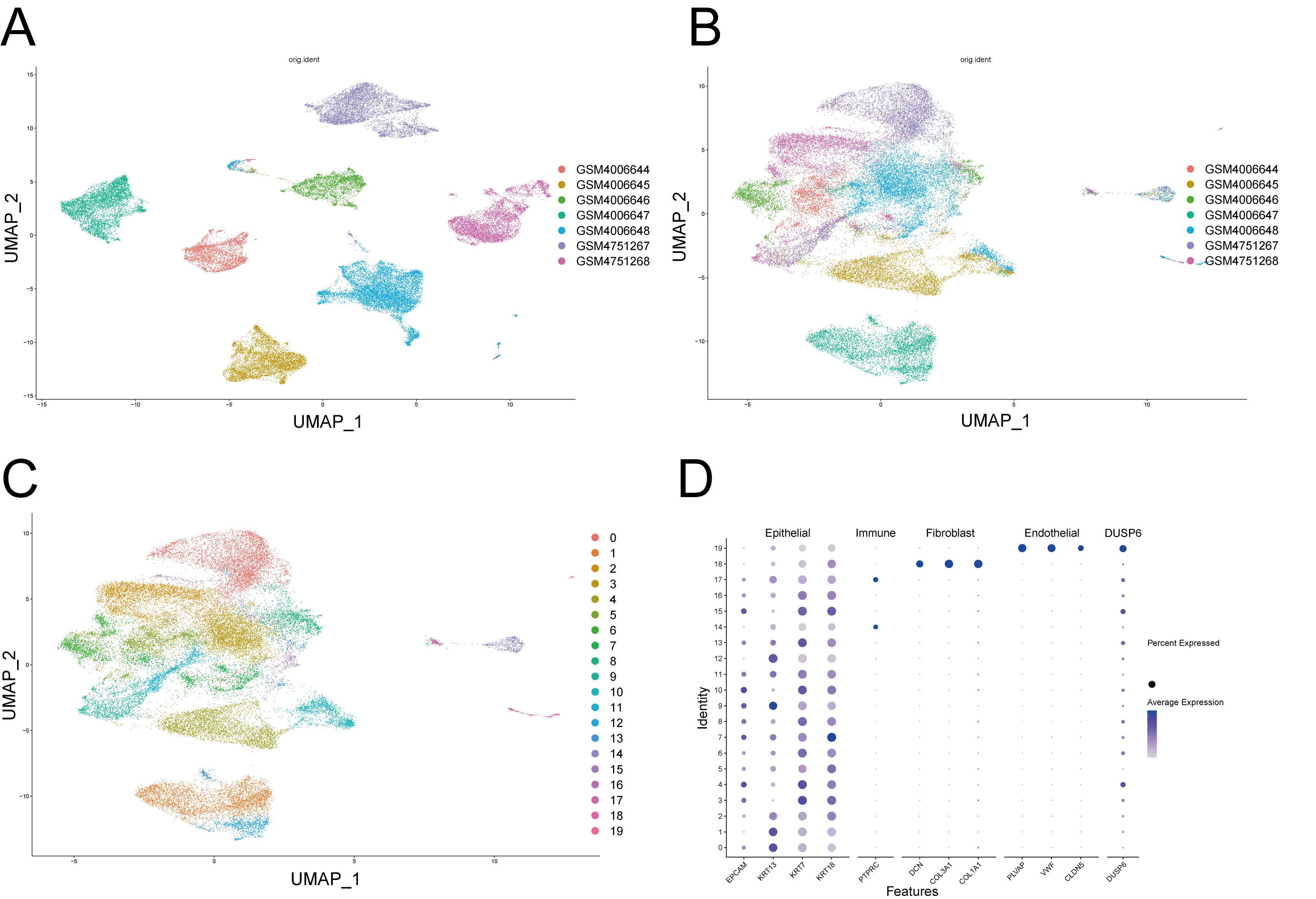

Supplement: Supplementary Figure 1 — (A) The UMAP plot before remove batch effect. (B) The UMAP plot of ‘harmony’ used to remove batch effect. (C) Cluster of scRNA-seq. (D) Dot plot show the marker genes and DUSP6 expression in every cluster. [file Image1.tif]

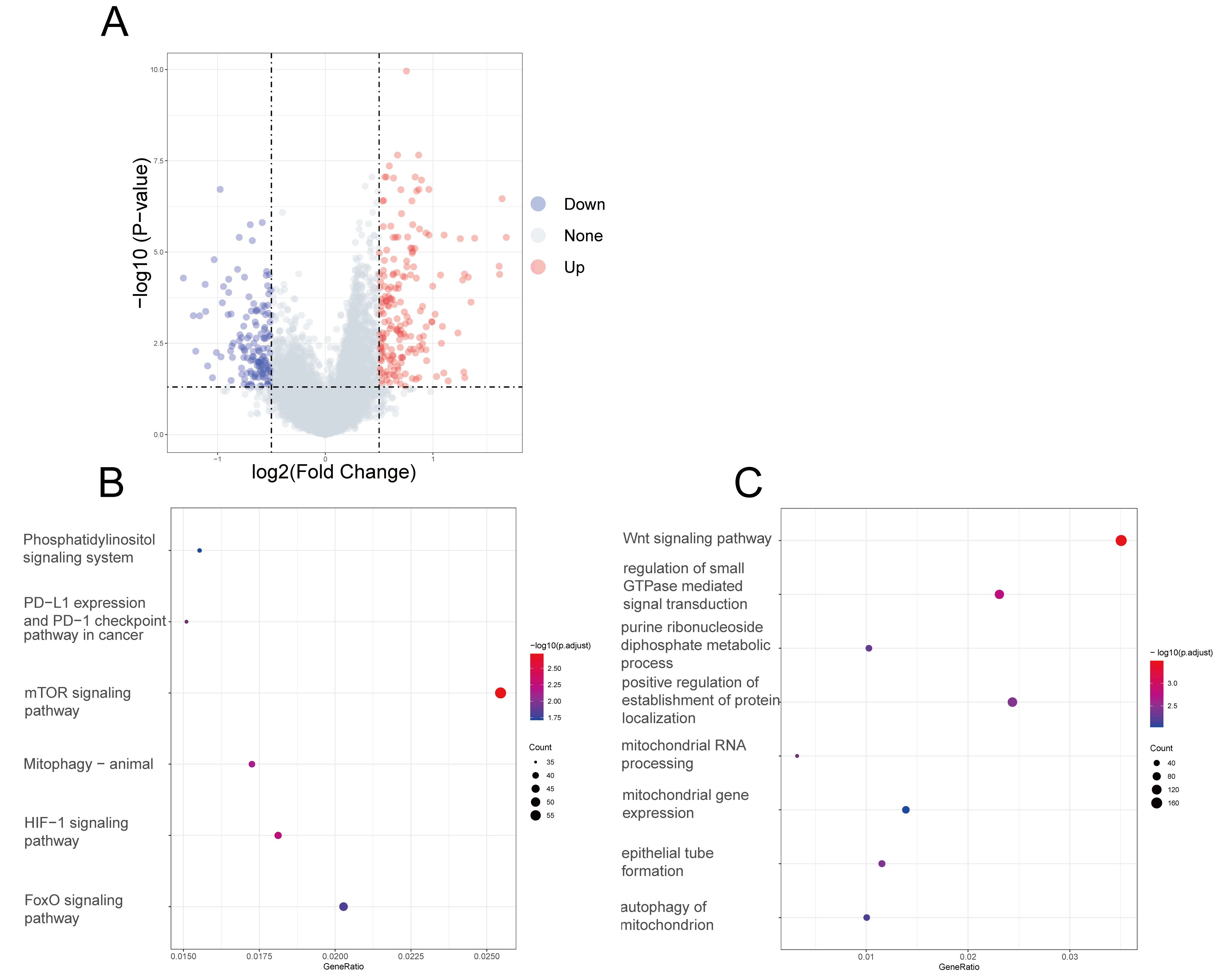

Supplement: Supplementary Figure 2 — (A) Differential expression genes between DUSP6 high and low group in TCGA-BLCA dataset (B) The KEGG enrichment analysis for differential genes was performed. (C) The GO enrichment analysis for differential genes was performed. [file Image2.tif]
